# Getting messier with TIDieR: embracing context and complexity in intervention reporting

**DOI:** 10.1186/s12874-017-0461-y

**Published:** 2018-01-18

**Authors:** Sarah Cotterill, Sarah Knowles, Anne-Marie Martindale, Rebecca Elvey, Susan Howard, Nia Coupe, Paul Wilson, Michael Spence

**Affiliations:** 1 Centre for Biostatistics, School of Health Sciences, University of Manchester, Manchester Academic Health Science Centre, Manchester, UK; 2 Alliance Manchester Business School, University of Manchester, Manchester, UK; 3 Centre for Primary Care, University of Manchester, Manchester, UK; 4 NIHR CLAHRC Greater Manchester, Manchester, UK; 5 Manchester Centre for Health Psychology, School of Health Sciences, University of Manchester, Manchester, UK

**Keywords:** Checklist, Reproducibility of results, Intervention fidelity, Research reporting standards

## Abstract

**Background:**

The Template for Intervention Description and Replication (TIDieR) checklist and guide was developed by an international team of experts to promote full and accurate description of trial interventions. It is now widely used in health research. The aim of this paper is to describe the experience of using TIDieR outside of trials, in a range of applied health research contexts, and make recommendations on its usefulness in such settings.

**Main body:**

We used the TIDieR template for intervention description in six applied health research projects. The six cases comprise a diverse sample in terms of clinical problems, population, settings, stage of intervention development and whether the intervention was led by researchers or the service deliverers. There was also variation in how the TIDieR description was produced in terms of contributors and time point in the project. Researchers involved in the six cases met in two workshops to identify issues and themes arising from their experience of using TIDieR.

We identified four themes which capture the difficulties or complexities of using TIDieR in applied health research: (i) fidelity and adaptation: all aspects of an intervention can change over time; (ii) voice: the importance of clarity on whose voice the TIDieR description represents; (iii) communication beyond the immediate context: the usefulness of TIDieR for wider dissemination and sharing; (iv) the use of TIDieR as a research tool.

**Conclusion:**

We found TIDieR to be a useful tool for applied research outside the context of clinical trials and we suggest four revisions or additions to the original TIDieR which would enable it to better capture these complexities in applied health research:

- An additional item, ‘voice’ conveys who was involved in preparing the TIDieR template, such as researchers, service users or service deliverers.
- An additional item, ‘stage of implementation’ conveys what stage the intervention has reached, using a continuum of implementation research suggested by the World Health Organisation.
- A new column, ‘modification’ reminds authors to describe modifications to any item in the checklist.
- An extension of the ‘how well’ item encourages researchers to describe how contextual factors affected intervention delivery.

**Electronic supplementary material:**

The online version of this article (10.1186/s12874-017-0461-y) contains supplementary material, which is available to authorized users.

## Background

Replicability and implementation of effective interventions is reliant upon accurate description of the intervention’s content and delivery. Indeed, it is known that the completeness of intervention description for non-pharmacological interventions is often lacking [1–4], which puts interventions at risk of being implemented incorrectly or not at all. In response to this, The Template for Intervention Description and Replication (TIDieR) checklist and guide [5] was developed by an international team of experts, to promote full and accurate description of interventions. TIDieR provides a standardised template for authors to describe all necessary elements for reporting of non-pharmacological interventions across 12 items (Table 1). Its development is linked to a wider movement towards standardising research reporting, illustrated by the growing EQUATOR network (Enhancing the QUAlity and Transparency Of health Research), which brings together access to 351 different reporting guidelines [6, 7] and has played a major role in new guideline development.

**Table 1:** TIDieR checklist^a^

TIDieR item	Brief description
1. Brief Name	Name or phrase describing the intervention.
2. Why	Rationale, theory, or goal of elements essential to intervention.
3. What (materials)	Physical or informational materials used, and where they can be accessed.
4. What (procedure)	Procedures, activities, and/or processes used in the intervention, including any enabling or support activities.
5. Who provided	Background, expertise of provider, and training given.
6. How	Modes of delivery, delivered to group or individual.
7. Where	Type of location.
8. When and How Much	Number of times, number of sessions, intensity and over what time period delivered.
9. Tailoring	What, why, when, and how of planned personalisation/adaptation.
10. Modification	What, why, when and how of intervention modification during study.
11. How well (planned)	If intervention adherence or fidelity was assessed, describe how and by whom, and if any strategies were used to maintain or improve fidelity.
12. How Well (actual)	If intervention adherence or fidelity was assessed, describe the extent to which the intervention was delivered as planned.

^a^Adapted from Hoffmann et al. 2014: Better reporting of interventions: template for intervention description and replication (TIDieR) checklist and guide [5]

Since its publication in 2014, TIDieR has been widely used to report interventions at various stages of the research process. It has most often been used within the context of randomised controlled trials, alongside the CONSORT statement [8] for reporting trial results, and the SPIRIT statement [9] for writing trial protocols. For example, Stevens et al. used TIDieR to ensure the standardised reporting of their back pain prevention intervention within the trial protocol [10]. Clear reporting of interventions at the protocol stage allows specification of the intervention prior to the start of a trial. TIDieR checklists are increasingly being included alongside the trial report, to provide a detailed account of the intervention and assist with implementation of the trial findings [11].

The quality of intervention description is important across many fields of health research. A detailed specification of the necessary components of an intervention makes it easier to determine what was actually implemented, allows replication in other settings or research studies, facilitates interpretation of the findings and clearly delineates two or more similar interventions from one another. Many journals, including the BMJ [12], BioMed Central journals [13] and Implementation Science [14] now insist that authors use appropriate reporting guidelines to accompany submitted manuscripts. Researchers already use CONSORT [8] when reporting randomised controlled trials and a recent addition is StaRI [15] for reporting implementation studies of complex interventions. Both CONSORT and StaRI explicitly recommend the use of a standardised checklist to describe the new intervention or services, and suggest TIDieR as an example.

The TIDieR authors envisaged its use outside the RCT context: ‘While the emphasis of the checklist is on trials, the guidance is intended to apply across all evaluative study designs’ ([5] p1), and this is starting to happen. Watson, Greening et al. [16] used TIDieR to provide a comprehensive description of a family-based childhood obesity intervention within their service evaluation, and report that by doing so they provide a clear account of any necessary modifications made during the implementation of the intervention. They advocate the use of TIDieR to ensure transparency of intervention reporting, which is essential to those involved in its implementation. The TIDieR checklist has been adapted for use within systematic reviews as a data extraction tool and to assess the quality of reporting of interventions [2, 3, 17–19]. TIDieR may require adaption to improve its ability to describe specific applied interventions, and TIDieR is currently being adapted to describe quality improvement interventions [18], educational interventions for training health professionals [20] and exercise interventions [21].

Complexity in health research is widely recognised [22], not only in terms of the traditional understanding of the evaluation of multicomponent interventions but increasingly of the complexity of the context (or system) into which interventions are placed [23]. This itself highlights the apparent tensions between fidelity of replication and the need to tailor interventions to be sensitive to different contexts [24]. Furthermore, context needs to be recognised as a process involving persons, resources, perspectives and activities, and not just a place. However, the role of adaptations and evolving mechanisms of action are recognised less often than static reports of ‘barriers and facilitators’ [25]. The capacity for formal reporting tools such as TIDieR to accommodate such factors has not previously been discussed, but it is clear that a means of effectively capturing and reporting the interactions of context and intervention delivery is essential if we are to design and evaluate interventions that can be effectively delivered in practice.

The aim of the paper is to reflect on our experience of applying TIDieR in six case studies, covering a variety of applied health research contexts, and debate its usefulness as a research tool outside the context of clinical trials.

## Main text

### Case studies – tidier templates

The TIDieR template was used as a tool for intervention description in six projects, summarised in Table 2. The interventions were: (i) A Diabetes Prevention Programme (DPP) for patients with a diagnosis of impaired glucose regulation (IGR), where blood glucose levels are above the normal range but are not high enough for a diagnosis of type 2 diabetes. The programme consisted of telephone behavioural and motivational support from a health advisor over 9 months (Telephone DPP) [26]; (ii) Primary care referral into diabetes prevention programmes: a nurse facilitator attended selected GP practices, searched the electronic records for patients at risk of diabetes, made appointments with patients to discuss their condition, and referred appropriate patients to a local diabetes prevention programme (GP referral DPP) [27]; (iii) Community referral into diabetes prevention programmes: a community organisation and a local authority health improvement team approached members of the public in community settings, completed diabetes risk scores, offered blood tests to those at risk of diabetes and referred eligible people to local diabetes prevention programmes (Community referral DPP) [27]; (iv) A commitment based intervention to promote behaviour change for weight loss among overweight adults attending weight loss groups in low socioeconomic areas (SMART-C booklet); (v) An audit and feedback intervention in primary care which implemented audit, health professional education and processes of care (such as medication reviews) to improve management of patients after an episode of acute kidney injury (AKI) (Primary Care Management of AKI Intervention) [28]; (vi) A primary care intervention to reduce the risk of harm from acute kidney injury (AKI) in people taking certain groups of medicines, by providing information on which types of medications to stop taking temporarily whilst ill (AKI sick day guidance) [29]. TIDieR descriptions of the six interventions are included in Additional file 1: Appendix 1. All the projects were undertaken by the National Institute for Health Research Collaboration for Leadership in Applied Health Research and Care Greater Manchester (NIHR CLAHRC GM) which is a partnership between providers and commissioners from the NHS (including Clinical Commissioning Groups (CCGs) and hospital trusts), industry, the third sector and The University of Manchester to help facilitate research getting into practice [30].

**Table 2:** Summary of the interventions included in this paper and the methods used to develop the TIDieR reports

Intervention/Study Features				TIDieR features	
Case Name	Clinical problem and intervention	Population, setting & service provider	Stage of Intervention^a^/Intervention led by	Participants in production or other form of stakeholder input (Voice).	Time point employed
(i) Telephone DPP	Telephone behavioural and motivational support for 9 months to help patients with IGR (impaired glucose regulation) to reduce their risk of developing diabetes.	Patients with IGR in the community. Health advisors and diabetes nurses (secondary care).	Feasibility in Context. Service led.	Research team members; Clinical team members (health advisors and diabetes nurses); commissioners (CCG and Trust leads); service leads; synthesis of qualitative research with stakeholders.	Iteratively during the project to capture the key elements, in collaboration with participants.
(ii) GP referral DPP	A nurse facilitator attended selected GP practices, searched the electronic records for patients at risk of diabetes, made an appointment with patients to discuss their condition, and referred appropriate patients to local diabetes prevention programmes.	Patients in primary care. GP practices. Nurse Practitioner.	Feasibility in Context. Service led.	Research team members; Clinical (nurse practitioner); commissioners (CCG and Trust leads); synthesis of qualitative research with stakeholders.	Iteratively during the project to capture the key elements, in collaboration with participants.
(iii) Community Referral DPP	A community organisation and the local authority health improvement team approached members of the public in community settings, completing diabetes risk scores, offering blood tests to those at risk of diabetes and referring eligible people to local diabetes prevention programmes.	Members of the public in community settings (churches, workplaces, markets). Two community organisations working in health promotion and screening with the public.	Feasibility in Context. Service led.	Research team members; commissioners (CCG and Trust leads); synthesis of qualitative research with stakeholders.	Iteratively during the project to capture the key elements, in collaboration with participants.
(iv) SMART –C Booklet	Goal setting and commitment intervention to improve behavioural/weight loss outcomes for people living in low socioeconomic areas who are overweight.	Members of the public attending local authority weight loss groups in low socioeconomic areas. Local authority staff (health improvement) delivering lifestyle interventions in the community.	Intervention development. Research led.	Research Team, with synthesis of content from multiple sources including a qualitative study with staff and service users and patient & public involvement.	Iteratively throughout intervention development process
(v) Primary Care Management of AKI Intervention	Management of patients who have had an episode of AKI (acute kidney injury) in primary care, using a targeted ‘audit and feedback’ intervention. Audits of hospital discharge summaries and primary care records were used to identify cases of AKI. Educational events about AKI were provided for primary care professionals. Processes of care including action planning, medication reviews, kidney function monitoring and communication with patients were implemented.	Patient who has had an episode of AKI and carers. GP Practice staff (GPs, nurses and practice pharmacists).	Intervention Development. Service and research led.	Research team; NIHR CLAHRC GM implementation team. Intention to be shared with other stakeholders during the project.	At the beginning of the project to inform the study protocol and implementation.
(vi) AKI Sick Day Guidance	Management of patients who have had an episode of AKI (acute kidney injury) in primary care, by providing a ‘sick day guidance’ alert card and verbal clarification from a health professional. The guidance informs patients taking potentially nephrotoxic medicines which ones to temporarily stop taking during a short illness (up to 48 h)	Patients taking medications known to lead to risk of AKI. Primary care settings: GP practices, community pharmacy.	Feasibility in context. Service led.	Research team only.	At the end of the project for reporting purposes.

^a.^The stage of intervention is defined according to a typology of NIHR CLAHRC GM projects developed by Ruth Boaden, Paul Wilson and Ruth McDonald, 2016 [31], which specifies five stages: Exploration (finding out what is going on), Explanation (explaining something new), Development (developing and implementing interventions), Feasibility in Context (implementation of an intervention previously developed somewhere else) and Exploitation (spread of an intervention into routine practice)

### Consensus workshops

Eight researchers involved in the six applied health research projects that had used the TIDieR templates met in two consensus workshops. The purpose of the workshops were to share information, identify any common themes arising from their experience of using TIDieR and to consider whether any changes were warranted for using TIDieR in such settings. During the first workshop the six cases were classified by clinical problem, population, setting, stage of intervention, leadership of the intervention and the time point that TIDieR was employed (Table 2). The stage of intervention was based on a NIHR CLAHRC GM typology of studies [31], which specifies five stages of research: Exploration (finding out what is going on), Explanation (explaining something new), Development (developing and implementing interventions), Feasibility in Context (implementation of an intervention previously developed somewhere else) and Exploitation (spread of an intervention into routine practice). Leadership, referring to whether the intervention originated with NIHR CLAHRC GM or was initiated by the services or stakeholders involved in delivery, was included to enable us to consider how the TIDieR was used across the different cases. The classification of the case studies was followed by an unstructured discussion on the experience of using TIDieR in the six applied health research projects. The findings of the first workshop were written up, and discussed again at a second workshop, at which the initial ideas were clarified and developed further.

The six case studies covered various clinical problems in population health and primary care, and settings across the community, local health authorities, and primary and secondary care services. TIDieR was used in different ways across the six cases. In the three diabetes prevention projects, TIDieR was a central part of the research design from the outset. We used the TIDieR checklist as a framework to structure a topic guide for 43 semi-structured interviews with key informants, purposively selected to include those involved in the conception, formation, management, design and delivery of these three interventions. We also examined relevant reports, journal articles, presentations, scripts and staff training materials to inform the TIDieR description. The data was used to produce a draft description of the intervention, which was circulated to research participants and other stakeholders for comment and subsequent revision. Our intention was to produce a thorough TIDieR description of the intervention, shared by researchers and those involved in the project. In one project (SMART-C booklet), the TIDieR template was used by researchers at an early stage to facilitate the design of a new intervention. It was used iteratively to compile relevant information and evidence from multiple sources (existing evidence, new qualitative data, systematic review and public/stakeholder involvement) for each main component of the intervention. In the two AKI projects, the template was used only within the research and implementation teams. In the AKI sick day guidance project the TIDieR template was compiled by researchers towards the end of the research process, based on their observations and data collection, with the intention of being able to summarise the intervention and describe it accurately in reports. In the AKI management project (which is ongoing at the time of writing) the researchers compiled the template during the early stages of the study, in conjunction with the implementation team. The template will be shared with other project stakeholders implementing the intervention, for comment and potential revision, during the study, as well as being used in the ways intended for the previous sick day guidance project. This approach is consistent with the findings discussed below which have indicated the value of joint input.

The cases represent a variety of interventions at the stage of ‘development’ or ‘feasibility in context’, both researcher and service led, and with the TIDieR employed at different time points and with different participants. We were therefore able to consider the use and utility of the TIDieR across a diverse sample.

### The complexities of using TIDieR for applied health research

We identified four themes which capture the difficulties or complexities of using TIDieR in applied health research: fidelity and adaptation, voice, communication beyond the immediate context, and use of TIDieR as a research tool, which are described in the results section. These are each discussed below. We also identified potential revisions or additions to the original TIDieR which would enable it to better capture and reflect key issues in applied health research, described in the discussion section.

#### Fidelity and adaptation

For health research to make a difference in the real world, beyond the confines of the immediate study, researchers must be able to report the internal and external contextual detail, local understanding and shift (defined as interventions evolving as they are implemented) at all stages of the research process. ‘This does not mean abandoning a rigorous definition of implementation in terms of completeness and ensuring high fidelity. It does, however, indicate that interventions need to be designed flexibly to allow tailoring to match the target-population characteristics and local resources’ ([32] p204).

We found TIDieR to be a useful tool to capture the implementation of interventions over time and encompass the messiness of research, by which we mean those changes and adaptations that occur over the course of projects as opposed to reporting a static intervention and neglecting the evolution of interventions in context. Item 11 of TIDieR reports any strategies designed to ‘maintain or improve fidelity’ ([5] p7) and item 12 describes how well fidelity was achieved. Though listed as separate items the authors note the relationship between the two; and state that complex interventions will necessitate more complex and comprehensive assessments of fidelity, such as paying attention to training, intervention delivery and reception. ‘This item (11)—and item 12—extend beyond simple receipt of the intervention (such as how many participants were issued with the intervention drug or exercises) and refer to “how well” the intervention was received or delivered (such as how many participants took the drug/did the exercises, how much they took/did, and for how long)’ ([5] p7).

Item 10 reports on modification, allowing consideration of what aspects were modified over time in this context, why when and how they were modified. For example, TIDieR was able to capture how, why and when the Community Referral DPP intervention changed from a focus on high risk communities (in terms of ethnicity and deprivation) to a more general population focus in response to perceived pressure from leaders for higher numbers of referrals. Using interview schedules guided by the TIDieR template, we elicited important information on how the staffing of the Telephone DPP service had changed over time to a service delivered solely by lay health advisors, whereas in the past there had been an initial phone call by a clinical diabetes nurse or dietician. In the AKI sick day guidance project the TIDieR clearly documented how useful facilitator observations were, as these illustrated not all practitioners had implemented the intervention as intended.

Though it is challenging to explore complexity in a table format, the authors of TIDieR have made efforts to engage with the dynamic messiness of research. The shift towards more nuanced, complex reflections on potential differences between the intended and experienced, or how it was understood and valued is welcomed. Making these habitual aspects of the research cycle clear within a summarised format has the capacity to enhance future interventions through highlighting areas for more detailed reflection using additional resources.

#### Voice

The purpose of TIDieR within the RCT setting is to enable researchers to clearly describe the interventions that are being tested, and those interventions are usually defined in the protocol at the outset. When TIDieR is used outside the context of a clinical trial, with an intervention that is being implemented by service providers and evolves over time, description becomes more complex. It becomes important to establish whose voice TIDieR represents: the research team, service deliverers or commissioners, or a combination. Effective and on-going communication between researchers and service providers, understanding of context, and building trusting relationships is linked to improved and sustained implementation [32]. As identified in Table 2, we took different approaches to the ‘voice’ when using TIDieR to describe the six interventions, dependant on the setting and context of each.

In the three diabetes prevention research projects we made a deliberate attempt to involve service providers in the development of the TIDieR intervention description. The inclusion of perspectives other than the researchers’ meant that the final tool reflected a more contextualised understanding of the interventions. In the Community referral DPP intervention, the service providers emphasised that the third sector organisation delivering the community intervention not only had prior experience of health promotion but had already developed relationships with the public health commissioners. The team was therefore able to capture these ‘soft’ ingredients (relationships, experiences, attitudes) that the service providers considered as essential professional background or expertise. In the AKI sick day guidance project the TIDieR template was written by the researchers, as a summary of the research findings, during the reporting phase of the study. The researchers found the template useful in producing a concise summary of the intervention and subsequently employed it from an earlier stage in the Primary Care Management of AKI project. This project is ongoing: at the time of writing a version of the template, completed by the researchers, had been included in the research protocol. The research team are planning to share the template with the project leads at the partnering organisation (a CCG) where the intervention is running, and potentially with providers, to prompt discussion about the intervention. The SMART-C researchers used the TIDieR template to identify where there were gaps in the evidence or where certain design elements were unclear. Initial design elements were identified from the existing literature and through a systematic review. A qualitative study which collected views of both service providers and service users, as well as researcher observations, which were then also included. Elements which were still unclear could then be clarified through further stakeholder feedback with service providers and staff, and through public involvement of service users.

On reflection, the inclusion of multiple voices is important because of the nature of applied health research, which is often concerned with evaluating interventions ‘owned’ by services or co-producing new interventions. Four of the six research projects were evaluations of pre-existing interventions that were designed or adapted by service leads (three diabetes prevention projects and the AKI sick day guidance). The other two (Smart- C booklet and Primary Care Management of AKI) involved co-producing new interventions between researchers and service leads. The nature of these projects meant that, unlike most trial contexts, researchers were reliant for their understanding of the interventions on seeking out the voices of those who designed them or those who would deliver and use them. In the single case where we didn’t do this it was due to lack of time, but has prompted the researchers to ensure they do this as the AKI sick day guidance study progresses.

This does however raise questions about how best, and to what extent, TIDieR can be used to adequately capture multiple voices and particularly to capture areas of disagreement. In the Telephone DPP study for example, the service leads reported that motivational interviewing was a component of the intervention but the research team felt that the content may not meet requirements to be reported as formal motivational interviewing and were reluctant to describe it as such. This proved helpful in this case, as the team were able to recommend to the providers certain criteria (such as independent review of staff interactions with patients and staff completing accredited training) that would need to be met if they wished to describe the intervention as motivational interviewing. Nevertheless this demonstrates that researchers and service providers may disagree about how the intervention can be described. The research team may still need to ‘own’ the document and have final say regarding its content, and ensuring that different perspectives are sought out and included should be part of a broader collaborative strategy rather than considered inherent to completion of the tool. To support this reflection, it may help to include a preliminary section in TIDieR detailing who had input into its production and the intended audience/audiences, including prompts to consider seeking input from participants at all stages of the development, implementation and adaption of the intervention.

#### Communicating beyond the immediate context

Increasingly, there is recognition of the need to support the spread of effective interventions and achieve operation at scale. To achieve this, mechanisms are needed for effectively summarising and distributing not only descriptions of intervention components but contextualised narratives of why and how such components were chosen and realised in practice. Therefore, a clear structured summary of an intervention, such as TIDieR, has potential utility beyond the immediate research context. The TIDieR template is a useful way of describing and documenting the intervention and its context, allowing replication or roll-out. The standardised format ensures that the key information is accessible, facilitating easier comparison between different interventions. Making explicit the drivers behind why elements were chosen (for example policy priorities or resource decisions) and the inclusion of ‘soft’ ingredients (such as the need for existing collaborations) can help future implementers to consider whether the same factors apply in their context, and anticipate what tailoring or adaptation may be required. The TIDieR description we developed for the Telephone DPP intervention was adopted by local service providers, who attached the TIDieR template to their application to UK government for continued funding.

However, we do not wish to imply that context can be captured through TIDieR as a simplistic and prescriptive feature of delivery. We do not think that context could or should be included as another ingredient or component that supports replication. Rather than context being part of the ‘recipe’ for delivery, its inclusion would be to ensure that in messy, complex situations in the real world, due attention is paid to how contextual factors have impacted on the intervention. This could help others interpret how their own contexts may impact on delivery, and consider broader barriers or facilitators at meso and macro levels. This could include for example whether there is professional capacity to deliver the intervention, pre-existing or planned local incentives to encourage uptake, and any existing local resource or capacity being drawn upon. For example, in the Community Referral NDPP, the research team explicitly commented that the organisations making referrals to diabetes prevention services had pre-existing established networks within local communities and community groups.

#### TIDieR as a research design tool

The process of using TIDieR to elicit the views of different stakeholders can expose disagreement about how the intervention is described and what constitutes its essential ingredients. As well as enabling the team to capture a more authentic picture of the messy reality of implementation in practice, this can highlight contested aspects of the intervention which may have an impact on effectiveness. In the Telephone DPP intervention, the iterative process of describing the intervention with stakeholders revealed tensions around moving delivery of the first steps of the intervention from the specialist diabetes team to community health advisors. Specifically, nurses and dieticians from the specialist diabetes team felt that their expertise was essential for this first step, compared to health advisors who felt their non-clinical skills could in fact be better suited to behavioural and motivational guidance work. Simply describing that the intervention was now delivered by health advisors would have masked the debate around necessary skills and expertise. Uncovering this debate prompted us to further qualitatively explore the decision making around the change of delivery and the perceptions of the staff involved regarding its impact both in terms of intervention availability (with specialist nurses being a more expensive and under demand resource) and effectiveness (with debate about the importance of clinical knowledge and experience compared to the motivational interviewing techniques employed by the health advisors). TIDieR can potentially be useful for facilitating stakeholders to reflect on what they are doing. It allows participants to see the whole picture of a service that they play a part in. In the SMART-C intervention, using TIDieR at an early stage provided a useful structure to identify what aspects of the intervention needed to be covered and where the gaps in evidence were, therefore ensuring all necessary elements of a complex and messy intervention were adequately addressed and described during the design process. The TIDieR template provided a streamlined documentation process for designing an intervention, particularly given that the evidence for the individual components of the intervention were drawn from multiple sources.

### Proposed changes to TIDieR

Overall, having used TIDieR for intervention description in six applied health research projects, we see it as a useful and valuable tool for describing the messiness of applied health research. Our research suggests changes to TIDieR, to enhance its utility for researchers working beyond the RCT, summarised in Table 3.

**Table 3:** Suggestions for additional items in TIDieR for use outside trials

Existing TIDieR item	Change	Description and explanation of change	Justification for addition
None	Addition of new item.	Who was involved in the preparation of TIDieR, how they were involved in the intervention/their perspective (e.g. researcher, service deliverer, patient, etc.)?	Increases transparency of reporting. Conveys whose voice the TIDieR description represents
	Voice: Whose voice does this description of TIDieR convey?		
None	Addition of new item	(i) What stage of implementation does the TIDieR checklist cover? (ii) Is this a revision of an earlier TIDieR checklist	Increases rigour of reporting. Conveys what stage the intervention has reached using a continuum of implementation research ranging from proof of concept studies through to those focused on implementation at scale and longer term sustainability [32].
	Stage of implementation		
10. Modification	Extension of current item.	Create a new column ‘Modification’ and remove 10. Modification	The column will remind people to describe modifications to all the TIDieR items.
	Modifications becomes a column rather than a row		
12. How Well (actual)	Extension of current item.	This change would encourage an explanation of the context of the implementation, and any adaptations made as a result. This will complement the Modifications column to convey change in implementation over time.	Understanding of intended and unintended consequences is important. Therefore an additional field to prompt inclusion of these elements might be a valuable addition for this purpose.
	Current text:		
	Describe the extent to which the intervention was delivered as planned		
	Suggested change:		
	Describe the extent to which the intervention was delivered as planned and outline the factors which had an impact on actual delivery.		

An additional item, ‘Voice’ would convey who compiled the TIDieR description and whose opinions were sought in its preparation. The addition of ‘Voice’ is not in itself a process recommendation that specifies how multiple perspectives can be included, and such a recommendation is likely to be beyond the scope of the template. Among our cases, there were different mechanisms employed for including multiple voices – directly seeking input on an initial version of a completed TIDieR template, synthesis and integration of qualitative research, and patient & public involvement input. Rather, it is intended as a prompt to encourage researchers to acknowledge if input from other stakeholders was sought or not. The TIDieR itself may then reflect multiple perspectives, but would not, for example, replace in-depth qualitative research in being able to fully capture differences in perspective. This would offer a means of explicitly incorporating insights from process-oriented qualitative data, demonstrating how they impact on delivery of the intervention itself (four of the six cases included qualitative data collection that informed the TIDieR). The issues of power and ownership however are complex and relate to interactions between the research team and the sites or people who are being researched. Explicitly prompting researchers to seek out other stakeholder views and capture or address differences could nevertheless provide a mechanism to encourage transparent reporting of these issues. It could also encourage researchers to be aware of issues locally that may impact on the implementation of an intervention.

An additional item ‘Stage of Implementation’ would convey what stage the intervention has reached, using a continuum of implementation research suggested by the World Health Organisation, which has stages ranging from proof of concept studies (is it safe and does it work?) through proof of implementation (how does it work in real-world settings?) to studies focused on implementation at scale and longer term sustainability [33]. Our experience with TIDieR suggests that all aspects of the intervention can by modified during the research period, so it would be useful to change ‘Modification’ to a column, to be completed for every item on the checklist. A useful extension to the ‘How Well (actual) item would go beyond simple description of the extent to which the intervention was delivered as planned, and explain the contextual factors that contributed to any modifications or changes that took place.

## Conclusions

We have used TIDieR for systematic intervention description in six research projects. Overall we found the tool beneficial for reflection, clarification and reporting, and we have identified four amendments to enhance its utility. These amendments illuminate the need for the explicit and dynamic reporting of research contexts, agents and voices, and their combined role on knowledge production; aspects of the research process which have previously been overlooked. The next steps in taking this forward would be a systematic literature review and A Delphi exercise to identify and reach consensus on additional TIDieR items for applied health research, following guidance developed by the EQUATOR network [6].

## Additional file

Additional file 1: Appendix 1: Completed TIDieR templates. (DOCX 58 kb)

## Abbreviations

AKI: Acute kidney injury
CCG: Clinical Commissioning Group
CONSORT: Consolidated standards of reporting trials
DPP: Diabetes prevention programme
EQUATOR: Enhancing the QUAlity and Transparency Of health Research
NIHR CLAHRC GM: National Institute for Health Research Collaboration for Leadership in Applied Health Research and Care, Greater Manchester
RCT: Randomised controlled trial
SPIRIT: Standard protocol items: recommendations for interventional trials
StaRI: Standards for reporting implementation studies
TIDieR: Template for intervention description and replication
UK: United Kingdom
WIDER: Workgroup for Intervention Development and Evaluation Research
