# Supplementary material for: Getting messier with TIDieR: embracing context and complexity in intervention reporting
Source: BMC Med Res Methodol. 2018 Jan 18;18:12. doi: 10.1186/s12874-017-0461-y (PMC5774137; doi:10.1186/s12874-017-0461-y)
Supplement: Additional file 1: — Appendix 1: Completed TIDieR templates. (DOCX 58 kb) [file 12874_2017_461_MOESM1_ESM.docx]

**Additional file 1**

**Appendix 1 - TIDieR templates for included projects**

**(i) Telephone DPP**

| **1. Brief name** | Salford IGR Care Call |
| --- | --- |
| **2. Why** | The goal of Salford IGR Care Call is to reduce the risk of diabetes in patients with a diagnosis of NDH, by use of regular telephone calls from a health advisor. The core elements of Salford IGR Care Call are:   1. Educational messages. Patients are initially given the [Leicester booklet](http://leicesterdiabetescentre.org.uk/Are-you-at-risk-of-Type-2-diabetes-Booklet), which helps people to understand why they are potentially at high risk of getting diabetes and provides ways to lower that risk. Educational messages continue to be delivered throughout the telephone follow up. 2. Goal setting and action planning (see appendix 3). It is about negotiating what the person wants to do, not telling them. 3. Health advisors are trained in motivational interviewing by the [Advancing Quality Alliance (AQuA)](https://www.aquanw.nhs.uk/) to help patients achieve/set new goals and form/review/maintain action plans. |
| **3. What – material** | ***1. Scripts***  The phone calls are guided by a series of scripts. Each script is on a different topic, but patients can prompt a return to a previous topic. IGR Care Call uses similar scripts to Diabetes Care Call, but with a focus on diet and exercise. The scripts were all written by a diabetes dietician or diabetes specialist nurse, based on evidence from the British Diabetic Association^[[1]](#footnote-1)^, NICE^[[2]](#footnote-2)^. The scripts have been updated annually by the diabetes team, to ensure they are based on best evidence, and a service level agreement (SLA) is being set up to ensure that continues (last update 2014).  ***2. Pathway***  The phone calls follow a pathway (see appendix iv).  ***3. Educational materials***  All patients receive the Leicester booklet prior to the first action planning call.  ***4. Patient records***  The telephone advisor has restricted access to an electronic patient record (Salford Integrated Record - SIR), so they can see diabetes-related test results and weight measures. They collect self-reported weights from the patients and add them to the record. This access is seen as very important to the success of Care Call, because the advisor can use this information in the conversation and can see, for example, if the person is currently in hospital. Having this information conveys to the recipient that the advisor is part of the NHS and can be trusted.  ***5. Written materials***  The health advisors have a stock of information leaflets they can send out to patients. These include: Home exercises, information on the Salford Community Leisure Active Lifestyles team, [Salford Heart Care](http://www.salfordheartcare.co.uk/) Healthy Heart Clubs (only for patients who are also diagnosed with a heart condition).  ***6. Information bank of resources***  Resources that can “help people implement their action plans”, for example local cookery classes, walking groups, education groups (including [PLANS](http://www.plansforyourhealth.org/) resources). The PLANS study^[[3]](#footnote-3)^ was delivered by the National Institute for Health Research Collaboration for Leadership in Applied Health Research and Care (NIHR CLAHRC GM), and led to the development of a [website](http://www.plansforyourhealth.org) that produces a personalised list of local activities including exercise, diet groups, social and hobby groups which support particular health and social needs. This also includes approved resources from various organisations, such as [World Cancer Fund](http://www.wcrf-uk.org/) information on portion sizes, and a [Diabetes UK](https://www.diabetes.org.uk/) shopping card on sugar content. In the past a DVD was produced and acted by a Salford dietician, and was sent to everyone to provide a visual representation of portion sizes. This is no longer used. |
| **4. What – procedures** | Patients are referred into the SRFT Diabetes Team by primary care or community teams.  ***Diabetes Team***   - Triage: check the necessary information has been provided (weight and BMI within one month; HbA1c result within six months of referral, to check on eligibility). If not provided, check the SIR or confirm with GP practice. The BMI and weight information is used to discuss targets for weight loss during the action planning call (APC). (NB. There is currently an additional stage of the pathway relating to recruitment to the [Comparison of Active Treatments for Impaired glucose regulation (IGR): a Salford Royal NHS Foundation Trust and Hitachi Collaboration](http://clahrc-gm.nihr.ac.uk/our-work/exploiting-technologies/catfish/) (CATFISH) trial. This is not described here as it relates to research administration rather than being part of the core service. - Introductory conversation and booking of the action planning call by a member of the administration team. - Leicester booklet sent out – this helps patients begin to think about areas of their lifestyle they may wish to address and how they might change. - Action planning call (30-40 minutes – with a nurse or dietician). There is guidance on what this should cover. Discussion includes: going through their diagnosis of NDH and what it means, their blood test results, going through their risk factors, confirming with the patient that they have read the booklet, encouraging the patient to identify things that they might want to change; ensuring the action plan is specific, measurable, attainable, relevant and timely (SMART). Calculate 5% and 10% weight loss targets. Importance and confidence rating on what you set out to do; supporting patients to adjust unrealistic goals. - Referred to Care Call by Diabetes Team for further calls; the agreed goals and action plans are passed onto Care Call, to use during the telephone calls.   ***Care Call Team***   - Telephone calls: Topics of discussion for each call (frequency described below in section 8) are ‘loosely’ planned in advance by the advisor (they are guided by the patient on the call so this isn’t prescriptively planned), based on: the individual’s goals and action plans; any measures from the patient record; choosing various standard scripts as appropriate. Advisors keep a record of what they have covered, so they can make sure, over time, they cover all the topics that might be useful with that patient. - Final call at nine months: Remind patients they should be called in by their GP for a repeat blood test at 12 months; symptoms of diabetes to watch out for; summarise progress. - Notify the GP of Care Call completion. |
| **5. Who provided** | The action planning call is currently offered by the specialist dieticians and diabetes specialist nurses in the Diabetes Team.  All other follow-up calls are delivered by trained non-clinical health advisors. There are five health advisors at present (4.5 FTE), all of whom have many years of experience of this work. They are currently managed by a qualified nurse who joined Care Call from the National Institute for Health Research Collaboration for Leadership in Applied Health Research and Care Greater Manchester (NIHR CLAHRC GM). The health advisors are recruited from many backgrounds, including office work and health improvement. The most important qualities are an excellent telephone manner, good communication skills, empathy, and understanding of long term conditions. All health advisors play the same role in the NDH service: there are no specialities.  Training: Health advisors receive at least two months in-house induction training, including: participation in three groups for people with T2DM: the [X-pert 6-week diabetes](http://www.xperthealth.org.uk/) patient education programme; education sessions for newly diagnosed T2DM patients; and education sessions for insulin starters). Advisors are given educational materials, take part in telephone role-play calls, observe diabetes clinics, attend medication training with the Diabetes Team, and listen to calls by fellow advisors. All the health advisors have received training in motivational interviewing provided through [AQuA](https://www.aquanw.nhs.uk/). |
| **6. How** | All the phone calls are made by the advisor to the patient. They are prearranged appointments. A patient has the same advisor throughout: this seems to be valued by patients, and builds trust. Information and health promotion resources are sent by post as required, tailored to individual needs. Calls are English language only. |
| **7. Where** | Care Call is part of the Salford Healthcare division of Salford Royal NHS Foundation Trust’s integrated care organisation. They are based in Summerfield House, in office accommodation. |
| **8. When and how much** | - The programme consists of nine calls; an introductory call, an action planning call, six calls over six months, and then a final call at nine months. The calls are set at monthly intervals. - The action planning call is 30-40 minutes in length. - Follow-up calls last around 10-20 minutes. Advisors are assigned 30 minutes per call, to allow time for preparation, posting out resources and record-keeping. - Advisors have capacity to make 12 calls per 7.5 hour working day. |
| **9. Tailoring** | There are a variety of scripts relating to NDH (including; portion control, weight loss, increasing activity, meal planning). The conversation is planned to meet the goals of the particular person, so is tailored, picking and choosing the scripts as guides on particular aspects of the conversation. Advisors emphasise flexibility and personalisation to individual circumstances to make material relevant and to foster a supportive relationship with the patient. They keep records on personal characteristics (disability, living situation) so they can adjust advice accordingly.  There is some flexibility to persist with patients who start to disengage. If a patient misses a call they are rebooked a new appointment. If someone misses two calls, they are discharged back to the GP, but if they re-contact Care Call at that point, they are accepted back onto the programme. |
| **10. Modifications** | ***Version IGR1*** *– 55 patients*   - Referrals from seven selected GPs, supported by NIHR CLAHRC GM. Action planning call (by Diabetes Team), and followed by six phone calls at monthly intervals (by Care Call staff, based in the Diabetes Team). - The evaluation demonstrated, (i) some patients (views were mixed) said they felt they would like a face-to-face or group session, and (ii) some patients felt abandoned after six months. In response to these concerns, version 2 of the service included the option of a group session at the start, and a final call at nine months.   ***Version IGR2*** *– 200 patients*   - Referrals from all GPs, supported by NIHR CLAHRC GM. Patients were given a choice of either (i) ½ day group education session (by Diabetes Team) (n=100), or (ii) action planning call (by Diabetes Team) (n=100) at the start, followed by six months of phone calls plus a final call at nine months (by Care Call staff, based in the Diabetes Team). - Current version – commissioned as mainstream service in April 2014, running since January 2015, with referral routes for both primary and community care providers. Primary care referrals received from GPs, with help from a nurse facilitator (funded by NIHR CLAHRC GM; Hitachi Europe Ltd; and the NHS DPP). Direct access referrals from community teams. Action planning call (by Diabetes Team) followed by monthly phone calls for six months, plus a final phone call at nine months (by Care Call staff now independent of the Diabetes Team). Education and action planning is provided by telephone to individual patients. There is no longer an initial face-to-face option.   ***Planned changes***   - Action planning call to be done by Care Call staff, instead of Diabetes Team. Care Call has historically been an integral part of the Diabetes Service, and Care Call continues to provide a diabetes support service to patients with type 2 diabetes. For patients with NDH, the service will in future be delivered wholly by the health advisors in the Care Call team to allow specialist resources to focus on patients diagnosed with diabetes. Diabetes nurses have previously undertaken the initial action planning calls with patients. Their role in reviewing the NDH scripts is currently under discussion. The Care Call staff are currently undergoing training to obtain the Royal Society of Public Health (RSPH) level 2 certificate in understanding behaviour change which will enable them to undertake action planning calls. They will also shadow the diabetes dietician who currently completes the action planning calls. - Interview respondents varied in whether they considered health advisors to be capable of delivering more formal action planning or whether this required more specialist (potentially clinical) expertise or experience. The health advisors themselves are confident about their ability to provide the full service (including the action planning call) in terms of skills, although they have some concerns about capacity. The primary drivers for reducing input from diabetes specialist nurses were the need to preserve limited specialist capacity for patients who have diabetes, enabling patients to connect with one worker throughout the whole process, and the appropriateness of health advisor skills to delivering behavioural or lifestyle focused interventions. - Some respondents suggested that continued provision of a group education session, as an alternative to the action planning call at the start, would have been helpful, to increase patient choice, cater to different patient preferences and to provide more visual information (for example around food portion sizes). The service does provide signposting to other group activities or support groups for those who wish to take these up. - IGR3, a blended service of online interactive health coaching materials developed by Hitachi Europe Ltd, in combination with telephone calls, is currently being trialled alongside Salford IGR Care Call. IGR3 provides patients with an online ‘dashboard’ to track their progress and communicate with their health advisor through a web interface. Depending on the results of the trial, this may be made available as a future option for patients. |
| **11. How well – planned** | The manager is located in the same office as those delivering calls, which may act as a form of quality monitoring. In terms of more formal monitoring, health care professionals such as the manager, diabetes dieticians and diabetes specialist nurses can listen into some of the calls. There is currently no facility to tape calls and listen to a random sample. Calls could then be evaluated for fidelity to the pathway, and against standard measures of motivational interviewing, such as [BeCCI](http://www.motivationalinterviewing.org/content/becci-manual)^[[4]](#footnote-4)^ or [MITI](http://www.motivationalinterviewing.org/content/miti-31)^[[5]](#footnote-5)^. |
| **12. How well – actual** | Care Call staff are enthusiastic and enjoy the job. Service managers reported that health advisors are well suited to their current role and can form effective relationships over the telephone. Telephone provision was considered to allow greater flexibility in contact with patients and also potentially enable a more honest dialogue to take place. The challenges relating to the action planning call are discussed above. |

**(ii) GP referral DPP**

| **1. Brief name** | Facilitated primary care referral into diabetes prevention programmes |
| --- | --- |
| **2. Why** | There was no specific theory or model described. The decision to set up the enhanced primary care referral route was made by commissioners to help achieve NDPP targets for recruitment into the local diabetes prevention services, based on the assumption that additional support for GP practices would improve referral rates. The commissioners accepted the offer from NIHR CLAHRC GM to fund a nurse facilitator to work in practices to increase awareness of the programmes and support GPs identifying and referring patients. Although locally GPs were already incentivised to do this as part of a long term conditions service, there was additional demand due to being part of the NDPP programme. |
| **3. What – material** | - FARSITE template – search criteria for practice database: FARSITE is a rapid ‘search and find’ tool, designed by North West EHealth, which allows searches to be run on anonymised population level health record data <http://nweh.co.uk/products/farsite>. If FARSITE was unavailable, this could be done using standard practice database searches. - Standardised letter sent to eligible patients from the facilitator (via FARSITE). - HITACHI leaflet explaining diabetes prevention, risk and interventions.   Standardised referral form to refer the patient into the Care Call service. |
| **4. What – procedures** | - The FARSITE tool is used to identify potentially eligible patients from the practice database. FARSITE searches were based on HbA1c result(s), age (18-90), excluding a diagnosis of diabetes. This search can be performed by staff within the practice or by the facilitator. - The facilitator reviews the list of potentially eligible patients, to exclude patients who would be unable to engage with the service, based on their patient record (e.g. patients who are deaf, have memory problems, cannot speak English, or who have a terminal illness). - The final list is reviewed by a member of the practice staff. Once the list is approved, all patients on the list are invited by letter to attend a clinic with the facilitator. This is done automatically through FARSITE. (Practices without FARSITE access would need to manually send the letters to patients.) - At the individual clinic appointment, the facilitator introduces themselves, and discusses the implications of the patient being at risk of diabetes and the importance of lifestyle change. They explain the service offered by Care Call and how it is delivered. [During the period of the research the nurse facilitator was also recruiting people for the Comparison of Active Treatment for Impaired Glucose Regulation (CATFISH) trial, a collaboration between Salford Royal NHS Foundation Trust and Hitachi]   If the patient expresses interest, the facilitator completes the referral letter to sends to Care Call. If they do not, they are provided with general health advice around diet and exercise. All patients are coded as IGR on the practice database. |
| **5. Who provided** | The facilitator was (and is recommended to be) from a clinical background, with some prior experience of diabetes prevention work. They must have access to the patient database, and therefore have appropriate governance approvals. They should be familiar with the service being offered and able to explain it fully. They should also be able to provide general health advice to patients who do not opt to be referred into the full service. |
| **6. How** | - The search can be conducted at the practice by the facilitator or by practice staff. - The consultation is a face to face one to one appointment between the patient and the facilitator. Appointments at the same practice are booked on the same day to provide a temporary clinic. |
| **7. Where** | Conducted at the practice surgery (requires a room). |
| **8. When and how much** | - During the research period the search is conducted once per practice. - The consultation with the patient is also a one-off. Currently no plans for the nurse facilitator to offer follow up appointments due to the short-term duration of the post (although follow up appointments can be completed by the practice themselves as part of routine work). |
| **9. Tailoring** | The criteria used to identify and exclude patients are standardised. There is no formal script for the consultation and it is likely to be tailored to an extent based on the individual. |
| **10. Modifications** | No modifications |
| **11. How well – planned** | No formal assessment has been conducted. |
| **12. How well – actual** | No formal assessment has been conducted. |

**(iii) Community referral DPP**

| **1. Brief name** | Community referral into diabetes prevention programmes |
| --- | --- |
| **2. Why** | There was no specific theory or model described. The decision to employ community identification was a commissioner-led decision based on reducing the burden on primary care services, increase appropriate referrals into existing IGR services and attempting to increase engagement with the wider Salford population. The outreach and engagement methods used were based on the previous experience and training of HIS and UI. |
| **3. What – material** | - Social marketing materials: these included eye catching materials for use in public (such as t-shirts and foam fingers), social media campaigns, and materials designed to instigate conversations with the public (such as offering free carrier bags at shopping centres) - Leicester Diabetes Risk Score^[[6]](#footnote-6)^: The score identifies people who may be at high risk of diabetes or currently have undiagnosed T2DM using data on age, sex, BMI, ethnicity, family history of diabetes and hypertension. - Portable point-of-care blood testing machines for use in the community (Afinion^[[7]](#footnote-7)^ machines were used in the pilot). - Referral form to Care Call service/Exercise for IGR. - Referral form to HIS if UI not co-located when a person is identified as potentially eligible. |
| **4. What – procedures** | - Members of the public are approached opportunistically and asked to answer selected questions from the Leicester Diabetes Risk Score, described to members of the public as a “lifestyle quiz”. This initial contact can also include height and weight measurements, depending on location and equipment available. Those scoring moderate to high (above 16) on the risk score are offered the opportunity to have a point-of-care HbA1c blood test. - If staff from the HIS have completed the risk score or if they are co-located with UI staff that have completed the risk score then this can take place immediately. If the HIS staff are not available then the UI team complete a referral form to arrange an appointment with a member of one of the HIS neighbourhood teams. - If the blood test shows the person to be eligible (score of 42-47 indicating high risk), they can be referred to the Care Call service or Exercise. If they score 48 or above they are referred to their GP, as this indicates diabetes. |
| **5. Who provided** | Salford HIS is part of Salford City Council. The HIS includes eight neighbourhood teams and a workplace advisor. They also have community volunteers and networks of existing community links (for example with local exercise and weight loss groups, Sheltered Housing). All staff have completed level 2 public health training. They also perform NHS Health Checks^[[8]](#footnote-8)^ and are qualified to perform point-of-care HbA1c blood testing.  UI includes both paid and voluntary staff. Volunteers have received training in engaging members of the public. All paid staff and a high number of volunteers have received local “Making Every Contact Count” training, and have training from UI in social marketing and brief advice focused on behaviour change. They have a focus on creative approaches and using local people to help network into communities. Volunteers working on this specific pilot were given training through UI on diabetes awareness and prevention.  Both services have experience of community engagement around health prevention and education, and existing networks of volunteers/community champions in the area. |
| **6. How** | Identification occurred face-to-face through staff members being located in community settings and opportunistically approaching members of the public. This was done both individually and in group settings (for example attending Weight Watchers group), although the risk score itself is completed on an individual basis. The blood testing and referral discussion are also conducted individually. |
| **7. Where** | - Public locations for opportunistic identification, for example supermarkets, shopping centres, religious centres such as temples, offices and weight-loss group meetings. - Targeted locations, utilising prior community networks of the organisations, for example in sheltered accommodation, religious centres, and exercise and weight loss groups. - Blood testing and waist circumference measurements are conducted privately if possible, although can be conducted in public. |
| **8. When and how much** | Individuals are identified and receive assessment once each, before being referred to diabetes prevention services. There is currently no recall service. |
| **9. Tailoring** | The questions asked are standardised. The specific process of engaging members of the public is adapted depending on the context and audience but involves opportunistic identification. |
| **10. Modifications** | No modiciations |
| **11. How well – planned** | Not applicable |
| **12. How well – actual** | No formal assessment of fidelity was conducted. However, compliance with the risk assessment requirements and completeness of the referral information is assessed in terms of whether the referrals are acceptable to the diabetes prevention services. Referral processes were refined and improved during the study. |

**(iv) Smart-C Booklet**

| **1. Brief name** | SMART-C Booklet |
| --- | --- |
| **2. Why** | ***Stage 1. Goal setting***   - Goal setting identified as ‘active’ component of weight loss intervention (Dombrowski et al, 2010). - People struggle to set realistic goals, goals often not set in group (QS).   ***Stage 2. Commitment making***   - Commitment making can improve outcomes, particularly to dietary goals (SR). - Lack of commitment/ pressure identified in group (QS)   ***Stage 3. Review goal***   - Goals often not revisited in groups, so don’t know if achieved or if need adjusting (QS) - There is a want/need for ongoing monitoring (QS) |
| **3. What – material** | Brief training manual provided for deliverers as requested (SF)  ***Stage 1. Goal setting***   - Booklet- Commitment Sheet. - Goals presented visually in the booklet using images as well as text, because of identified literacy/language barriers (QS) - Given pre-set goals because of difficulty setting realistic goals, and an option to set their own as requested by F (QS)   ***Stage 2. Commitment making***   - Booklet- Commitment Sheet. - Commitment in form of a behavioural contract; space for signature of SU and witness (F) below goal setting section (SR) in the booklet Commitment Sheets.   ***Stage 3. Review goal***   - Booklet- Review page - Tick boxes to identify if goal has been achieved or not. |
| **4. What – procedures** | ***Stage 1. Goal setting***   - F to provide booklet during 1^st^ session and talk through goal setting options. Must highlight that only one goal should be chosen (PPI). - Completed by SU, discussed with buddy (other SU within group), Supervised by F. Not recommended to choose spouse as buddy (SR)   ***Stage 2. Commitment making***   - SU and F to sign commitment before end of session (goal setting and signature is therefore witnessed- SR). This also gives the F the opportunity to check suitability of goal.   ***Stage 3. Review goal***   - F to review goals during individual weigh in or during group discussions (SF). |
| **5. Who provided** | - Facilitator must be familiar with health behaviour change and SMART goals. Training booklet provided (see above). |
| **6. How** | - Face-to-face delivery, in a group setting and in pairs to harness social support (QS) (Bukman et al, 2014) |
| **7. Where** | - Community venue where groups already run. |
| **8. When and how much** | ***Stage 1. Goal setting & Stage. 2 Commitment making***   - Minimum 6 weeks to tie in with current group (but could be ongoing- SUs want longer intervention QS) - Delivered once per session/week, lasting approximately 15 minutes.   ***Stage 3. Review goal***   - Previous weeks’ goal to be reviewed each week before new goal is set. To be done in pairs or during weigh in with facilitator (SF) |
| **9. Tailoring** | ***Stage 1. Goal setting***   - Goals can be chosen from the list or amended/ tailored to fit individuals’ needs (QS).   ***Stage. 2 Commitment making***   - n/a   ***Stage 3. Review goal***   - Goal can be tailored further here to make them achievable. Goals should then be adjusted dependant on review outcome. |
| **10. Modifications** | No modifications |
| **11. How well – planned** | - Fidelity will be addressed in the feasibility study. |
| **12. How well – actual** | - Adherence to be monitored by facilitator as part of ‘review’ section. |

**SR-** *Systematic review (under review)*

**QS** - *Qualitative study (Paper in preperation)*

**F** - *Facilitator*

**SU** - *Service User*

**PPI** - *Public involvement- Service users*

**SF** - *Stakeholder feedback- facilitators*

Bukman, A. J., Teuscher, D., Feskens, E. J., van Baak, M. A., Meershoek, A., & Renes, R. J. (2014). Perceptions on healthy eating, physical activity and lifestyle advice: opportunities for adapting lifestyle interventions to individuals with low socioeconomic status. BMC Public Health, 14(1), 1036.

Dombrowski, S. U., Sniehotta, F. F., Avenell, A., Johnston, M., MacLennan, G., & Araújo-Soares, V. (2010). Identifying active ingredients in complex behavioural interventions for obese adults with obesity-related co-morbidities or additional risk factors for co-morbidities: a systematic review. *Health Psychology Review, 6*(1), 7-32. doi: 10.1080/17437199.2010.513298

**(v) Primary Care Management of acute kidney injury (AKI)**

| **1. Brief name** | Bury Primary Care Management of AKI Intervention. |
| --- | --- |
| **2. Why** | Patients who have had an episode of hospital care complicated by AKI are at higher risk of worse health outcomes, including a further episode(s) of AKI, increased mortality, increased development or progression of CKD and high rates of hospital readmission. It is known that around two thirds of cases of AKI occur in the community, therefore, attention has focussed on improving the management of patients who have had an episode of hospital care complicated by AKI and been discharged. Following an episode of AKI, KDIGO guidelines advise testing kidney function after three months, whilst NICE recommends monitoring kidney function for two to three years; NICE also recommends that AKI risk is communicated with patients (and their carers) with a history of AKI. To date, evidence about the processes of identifying, recording and communicating about AKI in primary care has been scarce, therefore, the development and evaluation of a primary care AKI management intervention was warranted. ^[[9]](#footnote-9)^ ^[[10]](#footnote-10)^ ^[[11]](#footnote-11)^  Evidence exists for the effectiveness of targeted audit and feedback interventions, but these have not been used in kidney health, therefore, using a targeted audit and feedback intervention was warranted.^[[12]](#footnote-12)^  The intervention was designed to improve the management of care for people who have had an episode of hospital care complicated by AKI. |
| **3. What – material** | As part of the Bury CCG Quality in Practice Contract, general practices are required to participate in an audit of AKI activity, attend an educational event and develop a practice level action plan.^[[13]](#footnote-13)^  ***1. The audit***   - A manual ‘pre-audit’ provided information about patients in PAHT, with an episode of acute care complicated by AKI, between April 2015 and March 2016. The records of all general practices in the NHS Bury CCG area were audited to ascertain how many cases of AKI had been recorded in practices. Practices were then tasked with recording on their practice system, any cases that had not been recorded, using the relevant Read codes. - Following this, practices will be expected to record (code) all cases of AKI that appear on their patient’s discharge summaries; regular audits will be run to monitor the processes of care. NIHR CLAHRC GM and Vision will feed data back to practices. This will include the four processes of care outlined in 3 below, as well as data relevant to action plans developed by individual practices.   ***2. The education training sessions***   - A series of four training sessions was provided, which consisted of presentations on: AKI and its importance, a case study of AKI, the audit data, a demonstration of the Kidney Manager tool and a group session on action planning.   ***3. Action planning***  Development of a practice level action plan. In line with best practices, key processes suggested include:   - *Coding:* Has the AKI diagnosis been Read coded? In primary care, post-discharge. - *Medication Review:* Has the patient had a medication review within 4 weeks post discharge? - *Monitoring:* Has kidney function been checked within 3 months? - *Communication:* Has AKI risk been communicated to the patient (and carer)? (Documented through use of Read code 80AG) |
| **4. What – procedures** | ***1. The audit***   - A copy of the ‘pre-audit ‘results was provided to each practice, with instructions on how to record the outstanding cases, including which Read codes to use. - A tool – Kidney Manager - was installed onto practice systems, which ran in the background and was used to extract the audit data.   ***2. The education training sessions***   - Participants were provided with packs containing: the Read Codes for AKI, the Business Rules for the audit, a practice action plan template and ‘Think Kidneys’ literature on AKI. |
| **5. Who provided** | - The NIHR CLAHRC GM facilitator and project manager ran the manual pre- audit and visited practices to present the results. - The NIHR CLAHRC GM facilitator, project manager, programme manager and academic lead planned and facilitated the training events. The presentations were given by the CCG leads, the programme manager, project manager, academic lead, the renal consultant, and the Vision representative. - General practitioners, practice nurses, practice managers, practice administrators, practice pharmacists, medicines optimisation technicians participated in all stages of the intervention. |
| **6. How** | The pre-audit was carried out by the facilitator and project manager accessing hospital records. |
| **7. Where** | Following a data sharing agreement, the audit was based on hospital data from Pennine Acute Hospital Trust with further audits conducted and action planning and care processes implemented in general practices in one CCG area. |
| **8. When and how much** | The manual pre-audit was carried out once in summer 2016, the automated audit is running for 20 months (September 2016 to June 2018).  Participants attended one training event each; the events ran in November and December 2016.  Each practice developed one action plan, with a deadline of 31^st^ March 2017. |
| **9. Tailoring** | - Each practice was provided with their own audit results. - Each practice developed their own action plan, following the template. |
| **10. Modifications** | Unknown at present. |
| **11. How well – planned** | Unknown at present. |
| **12. How well – actual** | Unknown at present. |

**(vi) AKI Sick Day Guidance**

| **1. Brief name** | Salford Kidney Health Intervention Project / Salford sick day guidance service improvement initiative |
| --- | --- |
| **2. Why** | AKI is a syndrome that is common but preventable; initiatives with the potential to prevent AKI are now a high priority, particularly those involving self-care. Attention has focussed on the use of medicines ‘sick day rules’ or ‘sick day guidance’ as a way to avoid AKI. Sick day guidance instructs patients to stop taking medicines that can cause AKI, during episodes of acute illness that can result in dehydration, specifically when symptom(s) of vomiting, diarrhoea and/or fever are present. Sick day guidance for the prevention of AKI has been recommended in NICE guidelines, but there is little evidence that the approach reduces harm, therefore evaluation of a sick day guidance initiative was warranted.  The intervention was designed to reduce harm from AKI in people taking certain groups of medicines. |
| **3. What – material** | A list of resources, including guidelines and learning packages about AKI, was made available to providers. A pack was delivered to each provider site containing: Sick day guidance cards; an information leaflet and poster for providers with guidance on who to give the cards to and what advice to give; a promotional poster aimed at patients. The cards were credit-card sized and listed medicines that were to be stopped during an episode of vomiting, diarrhoea or fever, an outline of the relevant symptoms of illness and instructions on when to stop and re- start medicines (to stop if the symptoms were more than minor and to re-start when they were well). The text on the cards was replicated from the Highland sick day guidance card.  In addition, a ‘medicines optimisation detailed aid’ was produced for practice pharmacists  Sick day guidance cards were provided to patients. |
| **4. What – procedures** | Prior to implementation, two training events were facilitated by NIHR CLAHRC GM, with educational talks from other NIHR CLAHRC GM staff (the programme manager, the GP academic lead, a renal consultant from the local acute Trust and the CCG project lead. The training events provided information about AKI and about the sick day guidance initiative. NIHR CLAHRC GM facilitators visited all general practices and community pharmacies, to distribute the materials and to explain the project in person, subsequently, two visits were made to each site, to reinforce the project, offer support and provide additional materials.  Sick day guidance cards were provided to patients. Providers were encouraged to offer patients an explanation at the time that the card was provided, of what AKI is, why the card was relevant to them, in terms of which medicine they were taking and which symptoms were relevant. |
| **5. Who provided** | Healthcare professional and managerial staff were invited to the training events – although not all could attend these; the resources were delivered to all sites by the NIHR CLAHRC GM facilitators.  In phase one, general practitioners, health care assistants, practice nurses, community pharmacists and other pharmacy staff provided the intervention. During phase two, practice pharmacists also provided the intervention. |
| **6. How** | GPs, practice nurses, healthcare assistants and community pharmacists provided the cards during usual appointments or visits (to collect medicines from the pharmacy) with patients, to those prescribed a medicine(s) relevant to the intervention. The cards were provided to patients individually in person, cards were also put inside bags of medicine collected from pharmacies.  Practice pharmacists screened patient records to identify patients at higher risk of AKI - that is, those with a history of advanced stage CKD and/or previous episodes of AKI - and provide cards to patients, either in person, or by post with an accompanying letter, also to talk with patients, face to face or by telephone, to advise them about how to use the cards. |
| **7. Where** | General practices and community pharmacies in one CCG area. |
| **8. When and how much** | One card was provided to each patient, when they attended a general practice appointment or visited a pharmacy.  Practice pharmacists contacted patients who fit within their criteria for being at risk of AKI. |
| **9. Tailoring** | Whilst guidance on the explanation to give patients (described above) was provided, professionals were expected to use their professional judgement in deciding how to deliver the intervention. |
| **10. Modifications** | Piles of cards were noticed on pharmacy counters, which were available for anyone visiting the pharmacy to pick up and take.  Practice pharmacists encountered difficulties around the process of completing the record searches and communicating with patients in that there was not enough time to do this, consequently, no face to face appointments took place and pharmacists tried to contact patients by telephone.  One practice pharmacist developed their own AKI patient information sheet that was posted out with cards. |
| **11. How well – planned** | Adherence and fidelity were not formally assessed, however, the facilitation visits were designed to provide support and advice on delivering the intervention, and an understanding of how well the intervention was operating in practice was gained through these visits. |
| **12. How well – actual** | Practice pharmacists encountered barriers to obtaining the information they needed, for example they could not access hospital records. |

**The TIDieR (Template for Intervention Description and Replication) Checklist^[[14]](#footnote-14)^**

| **1. Brief name** | Provide the name or a phrase that describes the intervention. |
| --- | --- |
| **2. Why** | Describe any rationale, theory, or goal of the elements essential to the intervention. |
| **3. What – material** | Describe any physical or informational materials used in the intervention, including those provided to participants or used in intervention delivery or in training of intervention providers. Provide information on where the materials can be accessed (e.g. online appendix, URL). |
| **4. What – procedures** | Describe each of the procedures, activities, and/or processes used in the intervention, including any enabling or support activities. |
| **5. Who provided** | For each category of intervention provider (e.g. psychologist, nursing assistant), describe their expertise, background and any specific training given. |
| **6. How** | Describe the modes of delivery (e.g. face-to-face or by some other mechanism, such as internet or telephone) of the intervention and whether it was provided individually or in a group. |
| **7. Where** | Describe the type(s) of location(s) where the intervention occurred, including any necessary infrastructure or relevant features. |
| **8. When and how much** | Describe the number of times the intervention was delivered and over what period of time including the number of sessions, their schedule, and their duration, intensity or dose. |
| **9. Tailoring** | If the intervention was planned to be personalised, titrated or adapted, then describe what, why, when, and how. |
| **10. Modifications** | If the intervention was modified during the course of the study, describe the changes (what, why, when, and how). |
| **11. How well – planned** | If intervention adherence or fidelity was assessed, describe how and by whom, and if any strategies were used to maintain or improve fidelity, describe them. |
| **12. How well – actual** | If intervention adherence or fidelity was assessed, describe the extent to which the intervention was delivered as planned. |

1. Diabetes UK (2011). Evidence-based nutrition guideline for the prevention and management of diabetes [↑](#footnote-ref-1)
2. National Institute for Health and Care Excellence (2012). Type 2 diabetes: prevention in people at high risk. Nice guideline (PH38) [↑](#footnote-ref-2)
3. Blickem, C. et al. (2013). Linking people with long-term health conditions to healthy community activities: development of Patient-Led Assessment for Network Support (PLANS). *Health Expectations.* <http://clahrc-gm.nihr.ac.uk/wp-content/uploads/Blickem-PLANS-paper.pdf> [↑](#footnote-ref-3)
4. MINT – motivational interviewing. The Behaviour Change Counselling Index (BeCCI). <http://www.motivationalinterviewing.org/content/becci-manual> [↑](#footnote-ref-4)
5. MINT – motivational interviewing. Revised Global Scales: Motivational Interviewing Treatment Integrity 3.1.1. (MITI). <http://www.motivationalinterviewing.org/content/miti-31> [↑](#footnote-ref-5)
6. Gray LJ, Taub NA, Khunti K, et al. The Leicester Risk Assessment score for detecting undiagnosed Type 2 diabetes and impaired glucose regulation for use in a multiethnic UK setting. *Diabetic medicine : a journal of the British Diabetic Association*. 2010; 27: 887-95. [↑](#footnote-ref-6)
7. <http://www.alere.com/en/home.html> [accessed on 19/07/2016] [↑](#footnote-ref-7)
8. Gray LJ, Taub NA, Khunti K, et al. The Leicester Risk Assessment score for detecting undiagnosed Type 2 diabetes and impaired glucose regulation for use in a multiethnic UK setting. *Diabetic medicine : a journal of the British Diabetic Association*. 2010; 27: 887-95. [↑](#footnote-ref-8)
9. Kidney Disease: Improving Global Outcomes (KDIGO) Acute Kidney Injury Work Group. KDIGO Clinical Practice Guideline for Acute Kidney Injury. Kidney Inter., Suppl. 2012; 2: 1–138. [↑](#footnote-ref-9)
10. NICE Clinical Guideline [CG169]: Acute kidney injury: prevention, detection and management. August 2013 <https://www.nice.org.uk/guidance/cg169> [↑](#footnote-ref-10)
11. Wonnacott A, Meran S, Amphlett B, et.al 2014 Epidemiology and outcomes in community-acquired versus hospital-acquired AKI. Clin J Am Soc Nephrol 9(6):1007-14. doi: 10.2215/CJN.07920713 [↑](#footnote-ref-11)
12. Brehaut JC, Colquhoun HL, Eva KW, et. al 2016. Practice Feedback Interventions: 15 Suggestions for Optimizing Effectiveness. Ann Intern Med. 164(6):435-41. doi: 10.7326/M15-2248. [↑](#footnote-ref-12)
13. Appendix no. x Post-AKI Diagnosis Audit Business Rules 2016/17 [↑](#footnote-ref-13)
14. Hoffmann TC, Glasziou PP, Boutron I, et al. Better reporting of interventions: template for intervention description and replication (TIDieR) checklist and guide. *BMJ*. 2014; 7. [↑](#footnote-ref-14)
